# Selective Recovery of Europium and Yttrium Ions with Cyanex 272-Polyacrylonitrile Nanofibers

**DOI:** 10.3390/nano9121648

**Published:** 2019-11-20

**Authors:** Diego Morillo Martín, Leslie Diaz Jalaff, Maria A. García, Mirko Faccini

**Affiliations:** 1 Applied Chemistry & Materials, LEITAT Technological Center, C/Pallars, 179-185, 08005 Barcelona, Spain; dmorillo@leitat.org; 2 R&D Department, Leitat Chile, Román Díaz 532, Providencia, Santiago 7500724, Chile; ldiaz@leitat.cl (L.D.J.); magarcia@leitat.cl (M.A.G.); 3 Centro de Excelencia en Nanotecnología (CEN) Chile, Román Diaz 532, Providencia, Santiago 7500724, Chile

**Keywords:** water treatment, metal recovery, nanofibers, electrospinning, Cyanex 272, polyacrylonitrile, europium, yttrium, rare earth elements

## Abstract

Rare earth elements (REEs), which include lanthanides as yttrium and europium became crucial in the last decade in many sectors like automotive, energy, and defense. They contribute to the increment efficiency and performance of different products. In this paper nanofiber membranes have been successfully applied for the selective recovery of Eu(III) and Y(III) from aqueous solutions. Polyacrylonitrile (PAN) electrospun nanofibers were impregnated with a commercial organic extractant, Cyanex 272, in order to increase their affinity to rare earth metals ions. The coated nanofibers were characterized by SEM, ATR-FTIR, and TGA. Firstly, the adsorption of Eu(III) and Y(III) were evaluated in batch mode. Experimental data showed that the adsorption of Y(III) and Eu(III) corresponds to pseudo-second order model, with Langmuir sorption model being the best fit for both target ions. The results demonstrated that the adsorption capacity was high, showing a maximum capacity of 200 and 400 mg/g for Y(III) and Eu(III), respectively. Additionally, the presence of interfering ions does not show significative effects in the adsorption process. Finally, experiments in continuous mode indicated that the adsorption of the target elements is close to 100%, showing that PAN-272 is a promising material for the recovery of earth metal ions.

## 1. Introduction

The growing need for advanced materials and components, the global growth of population and natural resources depletion raises the question of whether there will be material supplies in the future. In that context, the so-called ‘critical raw materials’ (CRM) are crucial to meet today’s societal challenges and needs in many kinds of industries such as metallurgical, ceramics, electronics, and catalysis. They can be used in the production of materials like fluorescent lamps, lasers, super-magnets, turbines, thin film solar cells, atomic batteries, LEDs, high performance batteries, and cell phones [1,2,3,4]. Rare earth elements (REEs), which include the lanthanides, yttrium (Y), and europium (Eu), have been the focus of several studies in recent years due to their contribution in high-tech applications as luminescent materials, in the production of television screens, computer monitors, compact fluorescent light bulbs, X-ray scans, reactors, and lasers, among others [5,6]. The ever-increasing demand and limited resources for the extraction of these elements have motivated the development of new and sustainable methods for separation and recovery of REEs. The current recovery methods are precipitation [7], electrochemical methods [8], adsorption [9,10], solvent extraction [11,12,13], ion-exchange [14], and ionic liquid systems [15,16]. However, some of these technologies generate chemical contamination and require high energy consumption causing environmentally damages and increase in operational costs. On the other hand, chemical separation of trivalent lanthanides from the trivalent actinides is quite a challenge due to their similar chemical behavior and natural occurrence as a whole [17]. Therefore, it is desirable to produce suitable materials, prepared by scalable methods and that can selectively interact with trivalent rare earth metals. The development of a cost-effective and environmentally friendly alternatives for the recovery of REEs is crucial for a sustainable supply.

Liquid extractants have been widely employed to achieve selective recovery of REEs from water; however, they could generate secondary pollution as such products which are generally toxic and harmful for the environment must be used in large amounts. A good strategy to avoid this effect is the use of absorbent materials functionalized with specific and selective chemical groups. Membrane-based methods, especially those that are based in nanoscale fibers, have become a viable option due to the high porosity, permeability, and large specific area [18,19]. In that context, a very powerful and scalable method to produce nanofibers is electrospinning. This technique has been widely used in the last years because is relatively simple, economical, and versatile [20,21]. By selecting the optimal synthetic parameters, a wide variety of polymeric solutions could be used to prepare micro or nano materials.

Polyacrylonitrile (PAN) nanofibers has been recognized as a highly efficient material for the removal and recovery of metal ions from aqueous solutions. Furthermore, the introduction of appropriate chemical agent on the surface of the nanofibrous enables their utilization as a selective adsorbent for target elements or determinate compounds [22,23,24,25,26]. Cyanex 272, containing bis(2,4,4-trimethylpentyl) phosphonic acid as the main constituent, has been used to extract transition metals and lanthanides under appropriate conditions [27,28]. Diverse studies indicate that this extractant has several advantages including high selectivity, high separation factor and low aqueous acidity in extraction and stripping of rare earth ions [29,30,31,32].

The aim of this work is to prepare an adsorbent material based on electrospun nanofibers that can selectively recover Eu(III) and Y(III) ions from aqueous solutions. Polyacrylonitrile (PAN) nanofibrous membranes were then impregned with Cyanex 272 to combine the superior feature of the extractant with a nanostructured supported material, that can be easy reused, reaching high adsorption capacities using only small amounts of organic extractant. The effect of contact time, maximum loading capacity, and selectivity toward different interfering ions was first studied in batch mode. Then, the modified nanofibers were placed in a cartridge and tested in continuous flow mode for each target element. Although the separation of REEs using Cyanex 272 has been widely studied, to our knowledge, there are no reports on the extraction of rare earth metals by nanofiber membrane impregnated with such selective extractants.

## 2. Materials and Methods

### 2.1. Materials and Chemicals

Polyacrylonitrile (PAN, average molecular weight of 150,000), N,N’-dimethylformamide 99.9% (DMF), hydrochloric acid (HCl), sodium hydroxide pellets (NaOH), europium chloride hexahydrate (EuCl_3_·6H_2_O), yttrium nitrate (Y(NO_3_)_3_), zinc chloride (ZnCl_2_), nickel chloride hexahydrate (NiCl_2_·6H_2_O), copper chloride dihydrate (CuCl_2_·2H_2_O), cadmium nitrate tetrahydrate (Cd(NO_3_)_2_·4H_2_O), lead nitrate (Pb(NO_3_)_2_), nitric acid 70% (HNO_3_), phosphoric acid 85% (H_3_PO_4_), ammonium chloride (NH_4_Cl), sodium nitrate (NaNO_3_), ammonium sulphate ((NH_4_)_2_SO_4_), and ammonium dihydrogen phosphate ((NH_4_)H_2_PO_4_). All the reagents were used without further purification and were purchased from Sigma-Aldrich (St. Louis, MO, USA). Cyanex 272 (dialkyl phosphinic acid) was purchased from Solvay (Barcelona, Spain).

### 2.2. Synthesis of PAN Nanofibers by Electrospinning

The polymeric solution was prepared by dissolving PAN at 10 wt % in DMF and applying magnetic stirring overnight to obtain a homogeneous mixture. Commercially available electrospinning setup (NF-103, MECC Co. LTD., Fukuoka, Japan) was used to electrospun the PAN solutions. The procedure is described as follows: the polymeric solution was placed in a 20 mL syringe of 21 GA stainless steel needle attached to it. A power supply was used to provide a voltage of 25 kV between the syringe needle tip and a rotary drum collector. The operating conditions were; flow rate of 1.8 mL/h, working distance of 15 cm and rotational speed of 200 rpm. The electrospun fibers was collected on an aluminum foil obtaining a material of 60 × 20 cm size. The nanofibers were dried in a vacuum oven for 24 h at 60 °C for characterizations and adsorption experiments.

### 2.3. Impregnation of Electrospun PAN Nanofibers with Cyanex 272

In order to obtain a selective material towards Eu(III) and Y(III), electrospun PAN nanofibers were impregnated in a stable and homogeneous way, with the organic extractant Cyanex 272. This product is commercially available and is well known for its high selectivity for lanthanides. The experimental procedure is described as follows; 100 cm^2^ of PAN nanofibers were immersed in 100 mL of 5 wt % Cyanex 272 in toluene for 240 min at room temperature. Then, the modified nanofibers (PAN-272) were washed with toluene in order to remove the excess of Cyanex 272 and dried overnight at room temperature under a fume hood.

### 2.4. Characterization of PAN and PAN-272 Nanofibers

The nanofibrous mats were characterized using a scanning electron microscope (SEM, JSM-6010LV, Jeol Instruments, Tokyo, Japan). Each specimen was deposited in an aluminum holder and then coated with a 10 nm layer of gold (Cressington 108Auto, Cressington Scientific Instrument, Watford, United Kingdom). Diameters of no less than 50 individual fiber segments for a given fiber mat specimen were measured in the SEM images using with FibraQuant software 1.3 from which the average values were calculated.

Fourier transformed infrared coupled with attenuated total reflectance (ATR-FTIR, SPECTROM ONE, Perkin-Elmer, Waltham, MA, USA) analysis was carried out in a range of 4000–500 cm^−1^, with percentage of transmittance as measurement mode and 32 scans per sample with a resolution of 4.0 cm^−1^ to analyze chemical and physical interactions.

Thermogravimetric analysis (TGA, Q500, TA Instruments, Barcelona, Spain) was carried out to determine the polymer degradation temperatures in composite materials and quantify the amount of organic extractant coating the PAN nanofibers. The process was carried out by heating the samples with a heating rate of 10 °C/min until 900 °C under N_2_ atmosphere. The weight loss percentage versus temperature were represented.

### 2.5. Adsorption Experiments in Batch Mode

The adsorption experiments were performed in batch mode by mixing an aqueous solution of Eu(III) and Y(III) at room temperature with a constant mass of the PAN-272 nanofibers, using a rotary shaker. For all the experiments, the pH value was fixed to 3, being the pH where Cyanex-272 shows the maximum extraction performance [30]. The optimal pH was reached using standardized solutions of HNO_3_ 1.0 M or NaOH 1.0 M.

After mixing PAN-272 nanofibers with the target solution for the established time, the solid phase (adsorbent system) is removed from the solution and the Eu(III) or Y(III) composition was determinate by inductively coupled plasma mass spectrometry (ICP-MS, 7500cx, Agilent Technologies, USA). Additionally, phosphorus content was determined in every aqueous phase to determine Cyanex-272 loss during the adsorption process. The adsorption capacity for every system (*q*, mmol/g) was determined by using Equation (1)

(1) $$q=\frac{V_{ads}\cdot \left(C_{ini}-C_{e}\right)}{m_{ads}}$$

where *C_ini_* y *C_e_*are the initial and equilibrium concentration respectively (mmol/L), *V_ads_* is the volume of reaction (L) and *m_ads_* is the adsorbent mass (g).

To completely understand the adsorption process and the effects of external factors, different parameters were evaluated; the effect of the contact time with the target ions solutions, the maximum loading capacity and ionic selectivity towards interfering ions (common interfering cations and anions), the response of the material in adsorption–desorption cycles and the continuous mode experiments. The experimental details of every topic are presented below. All the tests were developed at constant pH and room temperature. The metal content in the supernatant was measured by ICP-MS and the anion quantity was measured by ion chromatography (IC).

#### 2.5.1. Effect of the Contact Time in the Adsorption Process

All the experiments were carried out by mixing 50 mL of 100 mg·L^−1^ Eu^3+^ and Y^3+^ solutions with a constant amount of the adsorbent material (100 mg). The contact time was varied in a range of 1-1440 min.

#### 2.5.2. Maximum Loading Capacity of the Adsorbent System

To determinate the maximum adsorption capacity of the impregned nanofibrous, a constant amount of the material was mixed with 50 mL of Eu(III) and Y(III) aqueous solutions with concentrations varying in the range of 1–1000 ppm. In every case the adsorbent was kept in contact with target solutions for 120 min.

#### 2.5.3. Selectivity Adsorption of Eu(III) and Y(III) in Presence of Interfering Heavy Metal Ions

The selective sorption of the target ions in presence of metal ions that are present in water or mineral lixiviates in high concentrations (Zn(II), Ni(II), Cu(II), Cd(II), and Pb(II) cations were investigated). The experiments were carried out by mixing 50 mL of 250 mg·L^−1^ of Eu(III) and Y(III) solutions mixed with the interfering aqueous solutions. The mixtures were in molar ratios of 1:0; 1:1, and 1:2 (target ion: interfering ion).

#### 2.5.4. Selectivity towards Most Common Interfering Anions

The selective sorption of Eu(III) and Y(III) in the presence of common interfering anions (Cl^−^, NO_3_^−^, SO_4_^2−^ or PO_4_^3−^) was investigated. The experiments were carried out by using 50 mL of 100 mg·L^−1^ solution of the target elements and with 0.25 M of interfering anions (molar ratio 400:1 respect of total metal content in solution). The experiments were performed as indicated before, by mixing a known amount of adsorbent systems with 50 mL of solution at room temperature for 120 min.

#### 2.5.5. Adsorption–Desorption Experiments

Firstly, desorption experiments were performed by using 10 mL of a stripping solution (HNO_3_, H_3_PO_4_, NaOH, or NaCl 1.0 M) that was added to PAN-272 nanofibers saturated with Eu(III) and Y(III). After 60 min of contact in a rotary shaker at room temperature, the aqueous and the solid phases were separated and the concentration of Eu(III) and Y(III) in the aqueous phase were determined by ICP-MS. Once the optimal stripping solution is selected, 4 adsorption–desorption cycles were performed. Adsorption experiments were carried out with 100 mg·L^−1^ solutions of Eu(III) and Y(III) for 60 min and the desorption experiments were carried out with 10 mL of the selected stripping solution. Experiments were conducted in duplicates.

### 2.6. Adsorption Experiments in Continuous Mode

**Figure 1:**
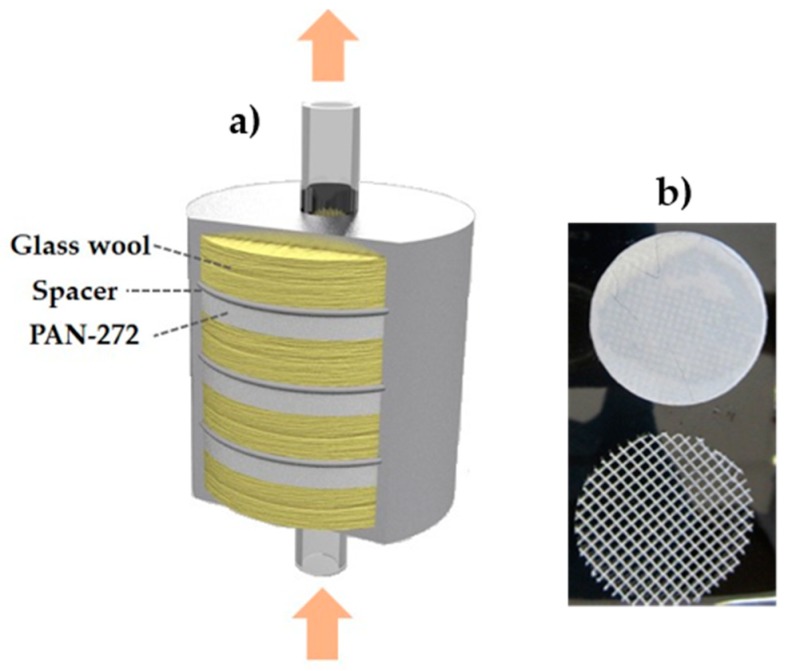
(**a**) Scheme of the cartridge filter design and (**b**) nanofiber membrane and spacer.

Adsorption experiments in continuous mode were performed to determine the behavior of the adsorbent system under practical working conditions. For this aim, a custom-made filtration metal cartridge of 6 × 4 cm was employed. A schematic representation of the system is shown in Figure 1. The cartridge is formed by PAN-272 nanofiber discs of 4 cm diameter with a polypropylene spacer placed between the membranes. Additionally, between each nanofiber-spacer pair a quantity of glass wool was introduced to fill up the cartridge.

Adsorption experiments were performed introducing 100 mg of PAN-272 nanofibers in the filtering cartridge and 1 L of 10 mg·L^−1^ Eu^3+^ or Y^3+^ solution was pumped through the cartridge with flow rate of 18 L/h for 6 h. An aliquot of the selected eluted solution was sampled periodically (0–360 min) to assess the concentration of the target ions. Experiments were conducted in duplicates.

## 3. Results and Discussion

### 3.1. Characterization of Electrospun PAN Nanofibers

#### 3.1.1. Scanning Electron Microscopy (SEM)

**Figure 2:**
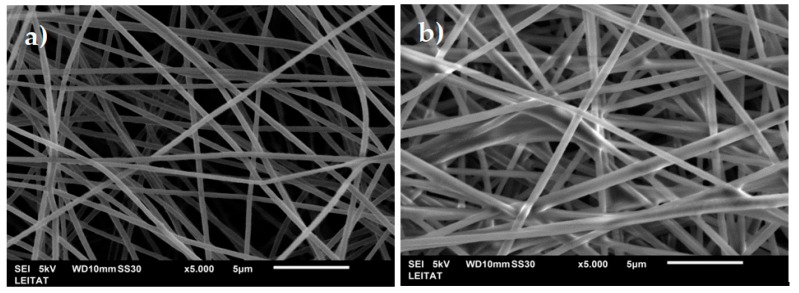
SEM images of (**a**) PAN unmodified and (**b**) PAN-272 nanofibers.

Figure 2 shows SEM images of the unmodified PAN nanofibers (PAN) and the impregnated nanofibers (PAN-272). As it can be seen, uniform structures were obtained without any identifiable beads, cracks, or defects. The average diameter of the nanofibers was 400 and 550 nm for the PAN and PAN-272 respectively. An increase in the diameter of the electrospun PAN nanofibers was observed after the impregnation process. This increase in size is an indication that the impregnation process has been carried out correctly. In both cases, randomly oriented nanofibers were obtained.

#### 3.1.2. Fourier Transformed Infrared Coupled ATR (ATR-FTIR)

**Figure 3:**
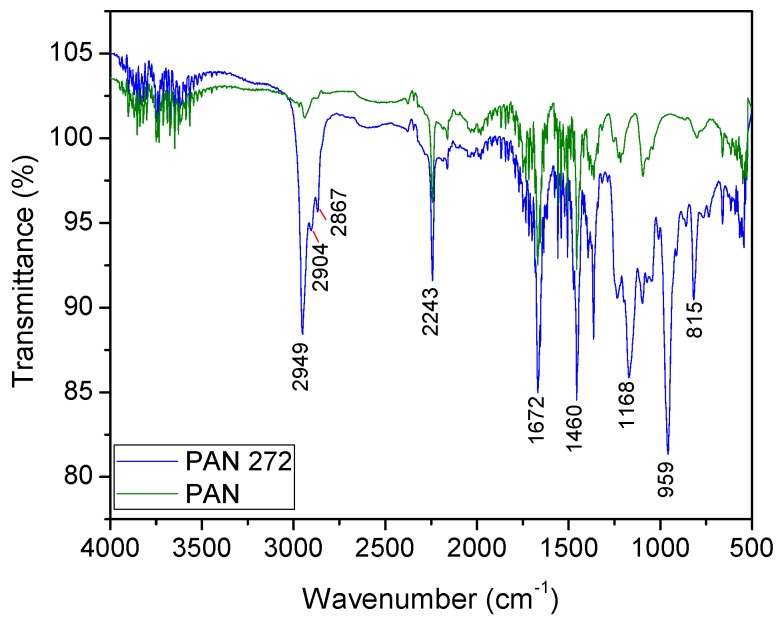
ATR-FTIR spectra of PAN and PAN-272 nanofiber membranes.

ATR-FTIR was used to identify the main functional groups of the modified and unmodified nanofibers. The spectra of PAN and PAN-272 are illustrated in Figure 3. CH-stretching (methyl, methylene) appears as a very strong band at 2950 cm^−1^, especially on the PAN-272 spectra due to effect of the incorporation of the organic extractant. Then, C–H stretching bands of symmetric and asymmetric vibrations and the bending vibration were observed at 2904, 2867, and 1460 cm^−1^ respectively [33,34]. The strong band at 2243 cm^−1^ correspond with the nitrile (–CN) of PAN nanofibers, observed in both samples as is expected [24]. The bonded OH vibration gives very weak and broad bands at 2700–2300 cm^−1^, whereas a sharp band centered at 1672 cm^−1^ represent OH deformation. A band centered at 1168 cm^−1^ is observed only in the PAN-272 due to the P=O stretching vibration, confirming the presence of Cyanex 272 in the membranes [35]. Finally, sharp bands centered at 959 and 815 cm^−1^ are assigned to P–O and P–O–C stretching vibration, that are characteristic of the organic extractant [35,36]. The results present a high homogeneity in the intensity for the different samples which indicate a homogeneous surface impregnation in the same sample and in the different modification batches.

#### 3.1.3. Thermogravimetrical Analysis

**Figure 4:**
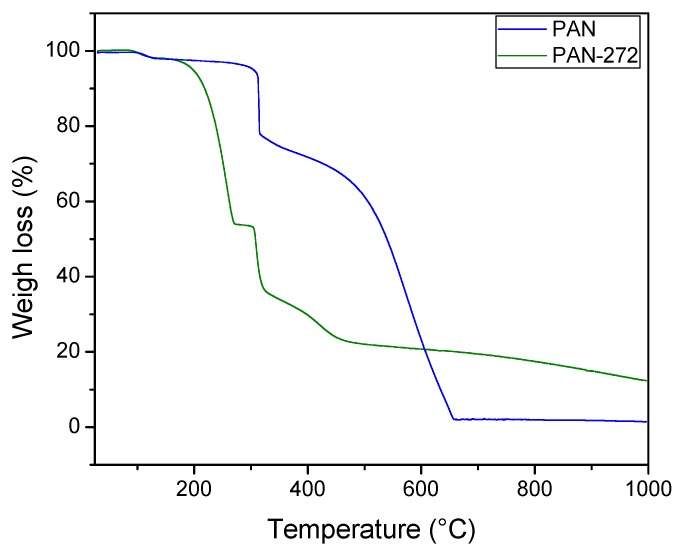
Thermogravimetric analysis of PAN and PAN-272 nanofibers.

Thermograms of PAN and PAN-272 samples are presented in Figure 4. The weight loss percentages were calculated using the first derivate of the thermogravimetric curves of pristine and coated PAN nanofibrous materials respectively. The results were presented in the Figure S1 and Table S1 of the Supplementary Materials. The thermal decomposition up to 150 °C for both samples was around 2% and it is attributed to the loss of water or solvent molecules on the nanofibrous membranes [34,37]. Then, a thermal event between 145–285 °C was observed only for PAN-272. The associated weight loss was 44.3% and it can be due the decomposition of Cyanex-272 coated on the polymeric nanofibrous. This event, together with the results shown in Figure 3 (FTIR), confirms the presence of the organic extractant in the polymeric nanofibrous membranes. The temperature interval is in agreement with the value reported for Kazak et al. [34]. A weight loss of 20.7 and 20.2 were observed in the range of 280–350 °C for PAN and PAN-272 respectively. Neisiany et al. [38] attributed this step to nitrile oligomerization, which produces volatile products, e.g., NH_3_, HCN, and CH_3_CN. The next step shown a weight loss of 75.9% was observed for the pristine PAN nanofibrous between 450–680 °C. This decomposition is associated with the complete decomposition of the PAN nanofibrous [37]. Instead, a two-step weight loss of around 10% was observed up to 350 °C for PAN-272. These thermal events correspond to an overlap of the decomposition of PAN nanofibrous and secondary products. Finally, the total weight loss of pristine PAN nanofibers is higher than PAN-272 (87.33%). This difference is expected due to the presence of phosphorous residual compounds.

**Figure 5:**
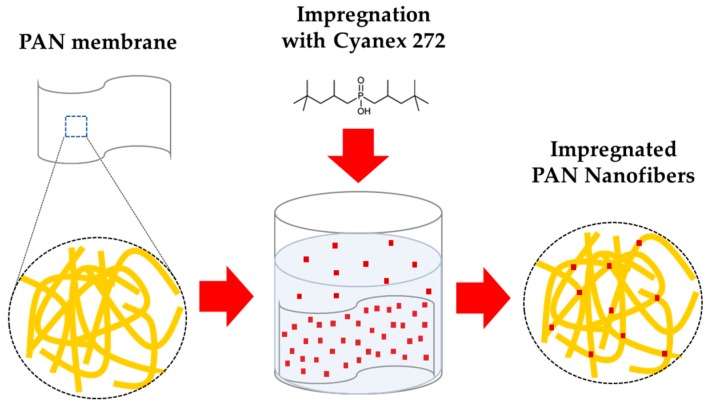
Schematic representation of the impregnation process on PAN nanofiber membrane. Red squares represent Cyanex-272 and yellow fiber represent the PAN membranes.

The characterization of PAN and PAN-272 confirms that the membrane is composed of nanofibers impregned with Cyanex-272. It is expected that carbon chains of Cyanex-272 interact with the PAN by hydrogen bonds, which makes that the Cyanex-272 were fixed in the nanofibrous. A schematic representation of impregnation process is presented in the Figure 5.

### 3.2. Adsorption Properties of PAN-272 Nanofibers in Batch Mode

#### 3.2.1. Effect of the Contact Time on the Adsorption Process

To evaluate the effect of the contact time in the adsorption process, an aqueous solution of the target elements Eu(III) or Y(III) was kept in contact with a constant quantity of the modified membrane (100 mg). The experimental conditions were described in Section 2.5.

**Figure 6:**
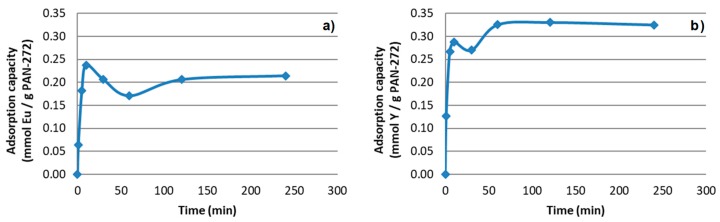
Adsorption of PAN-272 nanofibers for (**a**) Eu(III) and (**b**) Y(III) respectively

Pristine PAN nanofibers do not show any adsorption effect for both Eu(III) and Y(III). Therefore, the adsorption of the PAN-272 nanofibers can be attributed to the impregnated extractant Cyanex 272. The results of Eu(III) experiments are presented in the Figure 6a. At the beginning of the process, the metal adsorption is fast due the great affinity of Eu(III) and Y(III) for Cyanex 272, reaching an apparent maximum adsorption value in the first 10 min, probably due to a combination of chemical adsorption and other mechanisms such as physisorption. Then, a partial desorption was observed with a release of the target elements to the liquid phase until adsorption–desorption equilibrium is reached after 120 min with a constant value of 0.20 mmol Eu/g. On the other hand, experiments with Y(III) (Figure 6b) showed the same behavior reaching the maximum adsorption capacity and the adsorption equilibrium after 60 min of contact. In this case the metal uptake capacity at equilibrium was 0.33 mmol Y/g.

The maximum adsorption capacity difference between both target elements could be related with the difference in the atomic number between yttrium and europium. The stronger complexes with REEs are formed with increasing in the atomic number. However, this implies steric impediment, that is probably related with the difference in maximum adsorption capacity of target elements [39].

Additionally, the fitting to both pseudo-first and pseudo-second order kinetic models has been carried out and are presented in the Figure S2 (Supplementary Materials). The results showed that the first-order model does not fit well with the experimental data, while second order effectively represents the adsorption process with the time of the measurement. It can be concluded that the surface adsorption processes can be defined as two-site-occupancy adsorption mechanism.

#### 3.2.2. Maximum Loading Capacity of the Adsorbent System

**Figure 7:**
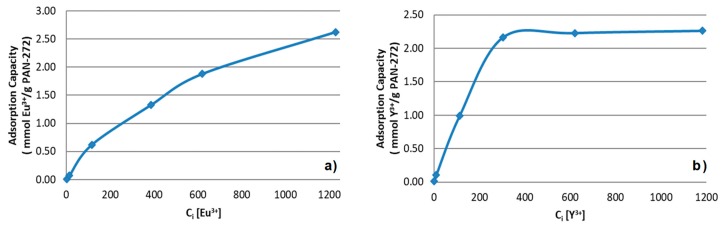
Effect of initial concentration of (**a**) Eu(III) and (**b**) Y(III) in the adsorption process using PAN-272.

The effect of the concentration of the target metals on the adsorption process were determined at its optimum pH value, a contact time of 120 min, room temperature and by varying the initial concentrations of Eu(III) and Y(III) from 1 to 1200 mg/L. The results are shown in the Figure 7. For Eu(III) experiments, an increase in the adsorption capacity is observed through all the measurement. The maximum loading capacity was 2.6 mmol of Eu(III)/ g. Meanwhile, for Y(III) an initial increase in the adsorption was observed until the system reach the saturation loading capacity at 2.3 mmol of Y(III)/g of nanofiber membrane.

In the Table 1, PAN-272 maximum adsorption capacities (*q_max_*) for Y(III) and Eu(III) in aqueous solution were compared with the values reported in literature for a wide variety of adsorbent systems under their optimal adsorption conditions. This benchmark demonstrates the superior efficiency of PAN-272 nanostructured adsorbent to uptake rare earth ion as it displays the highest uptake capacities for both Y and Eu among all materials. This exceptionally high adsorption capacity might be due to the large surface area of the PAN nanofibers combined with the Cyanex 272 molecules incorporated in the nanofiber surface, resulting in a larger number of chelating groups accessible for Eu(III) and Y(III) adsorption than other materials.

#### 3.2.3. Adsorption Isotherms

The two most widely used theoretical isotherms for the interpretation of the adsorption systems are Langmuir and Freundlich models. The Langmuir model is applicable for homogeneous adsorption systems as it is based on the assumptions of monolayer surface coverage and no interactions between adsorbed ions. For adsorption processes following Langmuir model, the adsorption capacity can be represented by [24]

(2) $$\frac{C_{e}}{q_{e}}=\frac{1}{q_{max}k_{L}}+\frac{C_{e}}{q_{max}}$$

where *C_e_* is the equilibrium concentration (mg/mL), *q_e_* is the adsorption capacity (mg/g), qmax is the maximum adsorption capacity (mg/g), and kL is the Langmuir dissociation constant.

Moreover, Freundlich isotherm is used for the description of heterogeneous adsorption. This model postulates that the adsorption energy of the metal ion depends on the occupation of the adjacent active centers. The adsorption capacity for Freundlich isotherm model can be calculated as [24]

(3) $$logq_{e}=logk_{F}+\left(\frac{1}{n}\right)logC_{e}$$

where *q_e_* is the adsorption capacity (mg/g), *C_e_* is the equilibrium concentration of target metal ions in solution (mg/mL), *k_F_* and n are the physical constants of Freundlich adsorption isotherm. These last terms, *k_F_* and *n*, are indicators of the adsorption capacity and adsorption intensity respectively. The Langmuir and Freundlich constants are presented in Table 2.

Langmuir and Freundlich plots were shown in the Figures S3 and S4 of the Supplementary Materials. The indicative parameters for every model are shown in the Table 2. The corresponding Langmuir plots of the experimental data gave a linear plot for both target metals on the adsorption process using PAN-272 nanofibers membrane. In both cases, the value of the determination coefficient (R^2^) is near to 1, indicating that the model fit well with the mentioned adsorption experimental data. Instead, the values of R^2^ for the Freundlich model were low, so we can conclude that the better adjustment for the adsorption of Y(III) and Eu(III) is the Langmuir model. The results obtained for adsorption isotherms indicates that the adsorption in both cases is homogeneous.

#### 3.2.4. Selectivity towards Most Common Interfering Ions

**Figure 8:**
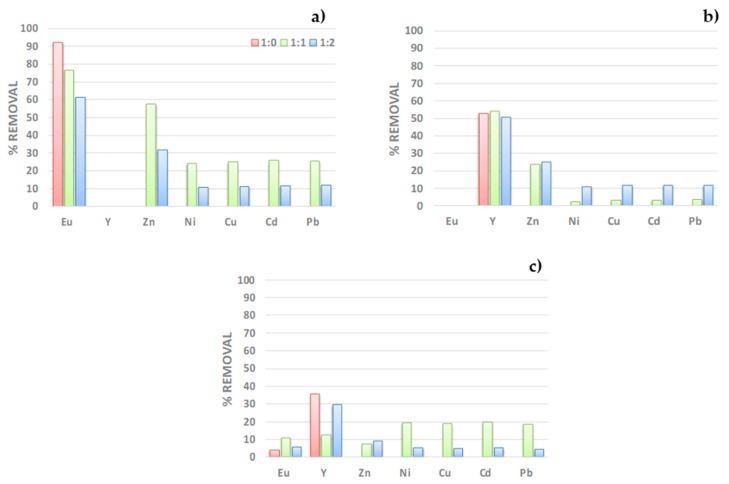
Removal percentage of (**a**) Eu(III), (**b**) Y(III), and (**c**) Eu(III)/Y(III) mixture in the presence of different interfering cations.

The experiments were carried out by mixing 250 ppm solutions of Eu(III) or Y(III) containing Eu (III), Y(III), Zn(II), Ni(II), Cu(II), Cd(II), and Pb(II) in molar ratios 1:0, 1:1, and 1:2 (ion of interest: interfering ions). The results displayed Figure 8, indicate that PAN-272 nanofibers can be used to selectively remove both Eu^3+^ and Y^3+^ from aqueous solutions, including in high ratios of interfering ions. When only one target rare earth ion is present in the solution, the percentage of removal is constant with and without interfering compounds presence of both target elements, Y(III) is recovered in higher amount than Eu^3+^ in a selective way. The repetitive tendency in a and c figures is indicative of a predominant effect of Eu(III) over Y(III), when a mixture of ions is made. This is interesting because may indicate a competition between the sorbates in aqueous solutions.

**Figure 9:**
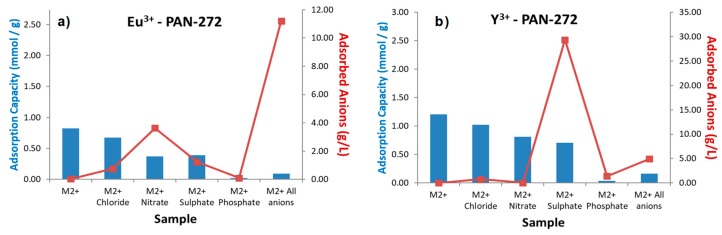
Selective adsorption of (**a**) Eu(III) and (**b**) Y(III) in presence of aqueous solutions of interfering anions with a concentration of 0.25 M (molar ratio 400:1 respect of total metal content in solution).

Finally, in the Figure 9 are presented the results of the selective adsorption of Eu(III) and Y(III) in the presence of common anions. The salts used to prepare the anions solutions were described in Section 2.1. The effect of anions in solution generates a low decrease in the adsorption capacity of PAN-272 nanofibers, being phosphate the anion with high interference in the adsorption process. Additionally, low adsorption of anions is observed which means that the nanofibers are negatively charged. In conclusion, and similarly to the metal ion selective nanofibers, although an interfering effect has been observed, higher adsorption capacity than other materials reported in literature has been achieved with each of the ion selective nanofiber developed [55].

#### 3.2.5. Adsorption–Desorption Cycles and Stripping Experiments

**Figure 10:**
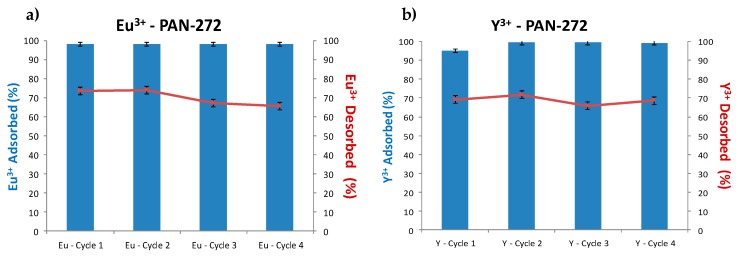
Adsorption–desorption cycles of (**a**) Eu(III) and (**b**) Y(III).

The adsorption–desorption experiments are shown in the Figure 10. As is seen in the bar graphics, the adsorption is almost constant, and it is around 100%. Moreover, the desorption cycles show constant desorption levels of 70% during 4 cycles, indicating that is possible to recover Eu(III) and Y(III), regenerate and reuse PAN-272 nanofibers for several cycles.

**Figure 11:**
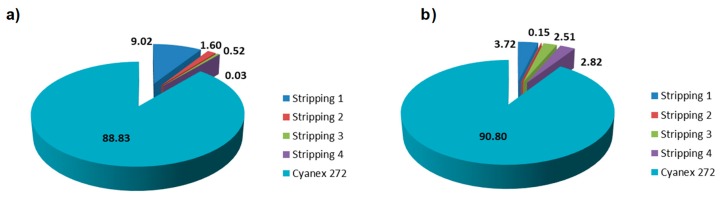
Cyanex 272 losses during the adsorption–desorption cycles and the remaining on the nanofiber surface for (**a**) Eu(III) and (**b**) Y(III).

Another important point to consider is the effect of the stripping solution on the release of chelating agent into the water solution, thus generating a secondary contamination. For this aim, the presence of Cyanex 272 (phosphorous content) in the water was quantified during the adsorption–desorption cycles. As shown in the Figure 11, nanofibers could retain around 90% of the Cyanex 272 that is impregnated into the fibers after 4 stripping cycles. This result means there is a strong interaction between the PAN nanofibers and Cyanex 272 chains that provides stability and fixation of the Cyanex 272 agent.

### 3.3. Adsorption Properties of PAN-272 Nanofibers in Continuous Mode

**Figure 12:**
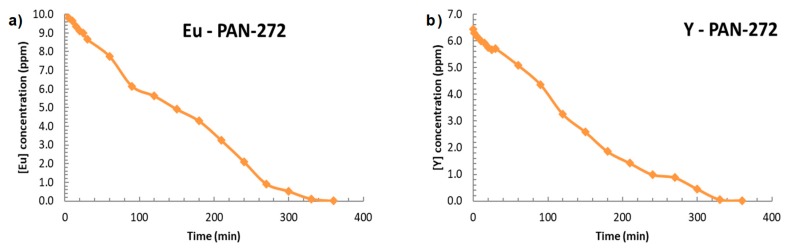
Adsorption in continuous mode of PAN-272 nanofibrous for (**a**) Eu(III) and (**b**) Y(III).

Finally, the Eu(III) and Y(III) adsorption in continuous mode is presented in the Figure 12. 1-L solution with 10 mg/L of Eu(III) or Y(III) solution was pumped throughout a multilayer cartridge containing PAN-272 nanofibers, disposed as is shown in the Figure 1. In both cases, the adsorption is close to 100% after 360 min reaching adsorption capacities of 0.97 mmol Eu(III)/g and 1.06 mmol Y(III)/g which confirms that PAN nanofibers membranes impregnated with Cyanex 272 are capable to retain Eu(III) and Y(III) with high efficiency.

## 4. Conclusions

In summary, a novel ion selective nanostructured adsorbent with high uptake capacity for Eu(III) and Y(III) was developed by loading PAN nanofibers with Cyanex 272 by impregnation process. The resulting material (PAN-272) was fully characterized by SEM, ATR-FTIR, and TGA and its performance was fully studied both in batch and in continuous operating modes. The maximum adsorption capacity in batch mode was 2.3 and 2.6 mmol/g of nanofiber for Y(III) and Eu(III), respectively. Additionally, the adsorption results demonstrated that PAN-272 present selectivity for the recovery of Eu(III) and Y(III) even in a high ratio of interferents (cations or anions), with phosphate being the major interferent for the adsorption process. Moreover, the impregnated nanofibers present good adsorption results after 4 adsorption–desorption cycles. Stripping experiments demonstrate that Eu(III) and Y(III) can be recovered employing acid solution with high recovery rates (>70%) while retaining around 90% of the organic extractant loaded into the nanofibers. Finally, PAN-272 nanofibrous material showed exceptional adsorption capacity, being able to recover almost 100% of target rare earth ions from water solution after 120 min in continuous operation mode.

## Figures and Tables

**Table 1:** Adsorption capacity comparison between different adsorbent systems

Y(III)			Eu(III)		
Adsorbent	*q_max_* (mg/g)	Ref.	Adsorbent	*q_max_* (mg/g)	Ref.
PAN-272	200	This work	PAN-272	400	This work
Alginate	181.8	[40]	Opuntia ficus indica cactus fibers	72	[41]
Nano-thorium(IV) oxide and nano-zirconium(IV)oxide	10–18	[42]	Barium carbonate	16	[43]
Maghemite	13.5	[44]	Zr and Ti phosphates	20–50	[45]
Ferric hydroxide	N/R	[46,47]	Kaolinite	1.2	[48]
Boron suboxide	N/R	[49]	T. Conoides (alga)	138.2	[50]
Montmorillonite	N/R	[51]	Chitosan microparticles	375	[52]
			Al-substituted goethite	6.75	[53]
			Palygorskite	24.26	[54]

N/R: Not reported.

**Table 2:** Langmuir and Freundlich constants and correlation coefficients

Target Metal Ion	Langmuir Constants			Freundlich Constants			
	*q_max_* (mg/g)	*k_L_* (L/mg)	*R^2^*	*q_max_* (mg/g)	*k_F_*	*n*	*R^2^*
Y(III)	200	0.2564	1	380	17.697	2.24	0.8402
Eu(III)	400	0.0646	0.9879	396	92.2996	4.24989	0.6825

## Supplementary Materials

The following are available online at https://www.mdpi.com/2079-4991/9/12/1648/s1; Figure S1. Thermograms and the first derivate of PAN and PAN-272; Figure S2. Fitting experimental data for first and second order kinetic models for Y(III) and Eu(III); Figure S3. Fitting experimental data for Langmuir and Freundlich models for Eu(III) and Y(III); Figure S4. Langmuir and Freundlich models fitted graphs for Eu(III) and Y(III); and Table S1. Weight loss of PAN and PAN-272 nanofibers membranes.
