# Supplementary material for: Selective Recovery of Europium and Yttrium Ions with Cyanex 272-Polyacrylonitrile Nanofibers
Source: Nanomaterials (Basel). 2019 Nov 20;9(12):1648. doi: 10.3390/nano9121648 (PMC6955798; doi:10.3390/nano9121648)
Supplement: Supplementary file 1 [file nanomaterials-09-01648-s001.pdf]

Supplementary Materials

Selective Recovery of Europium and Yttrium Ions with Cyanex 272-Polyacrylonitrile Nanofibers

Diego Morillo Martín [1], Leslie Diaz Jalaff [2,3], Maria A. García [2,3], and Mirko Faccini [1,2,*]

[1] Applied Chemistry & Materials, LEITAT Technological Center, C/Pallars, 179-185, 08005 Barcelona, Spain, dmorillo@leitat.org

[2] R&D Department, Leitat Chile, Román Díaz 532, Providencia, Santiago 7500724, Chile; ldiaz@leitat.cl (L.D.J.); magarcia@leitat.cl (M.A.G.)

[3] Centro de Excelencia en Nanotecnología (CEN) Chile, Román Diaz 532, Providencia, Santiago 7500724, Chile

* Correspondence: mfaccini@leitat.org; Tel.: +34-93-788-2300; Fax: +34-93-789-1906

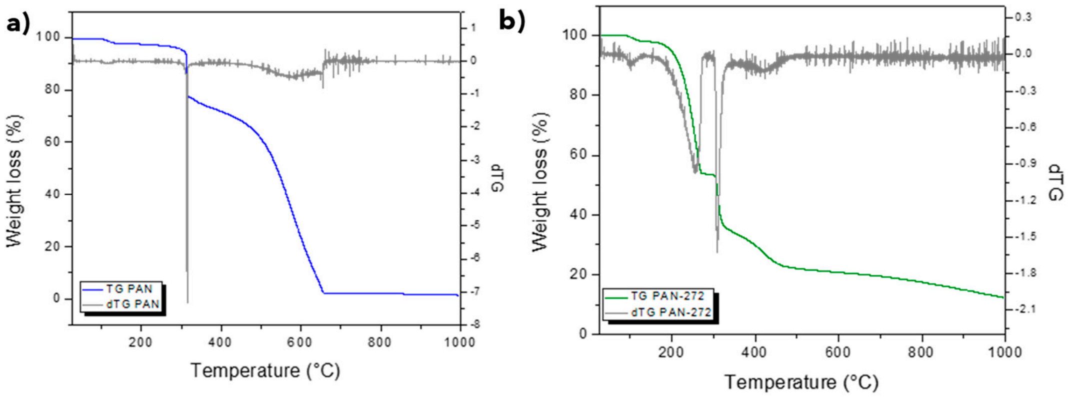

Figure S1. Thermograms and the first derivate of PAN and PAN-272

Table S1. Weight loss of PAN and PAN- 272 nanofibers membranes

Temperature range (°C)	PAN-272(%)	PAN (%)
<150	2.08	1.7
145-284	44.3	---
280-350	20.2	20.7
350-450	10.9	75.9
>450	10.0	

Kinetic fitting

The kinetic model of pseudo-first order has been widely used to predict adsorption kinetics. The model given by Lagergren is defined as:

$$q_t = q_e\,(1 - e^{-k_1 t}) \qquad \text{(S1)}$$

Integrating equation (S1) with respect to the boundary conditions $q_t = 0$ to $t = 0$ and $q_t = q_e$ to $t = t$:

$$\log(q_e - q_t) = \log q_e - \frac{k_1}{2.303}t \qquad \text{(S2)}$$

where k_1 is the pseudo first order constant (min^{-1}); q_t and q_e are the adsorption capacities of the ion-selective nanofibers at time t and at equilibrium respectively and t (min) is the contact time.

The pseudo second order equation based on equilibrium adsorption is expressed as:

$$q_t = \frac{q_e^2 k_2 t}{1 + q_e k_2 t} \qquad \text{(S3)}$$

Integrating equation (3) with respect to the boundary conditions $q_t = 0$ to $t = 0$ and $q_t = q_e$ to $t = t$:

$$\frac{t}{q_t} = \frac{1}{q_e^2 k_2} + \frac{t}{q_e} \qquad \text{(S4)}$$

Where k_2 is the pseudo second order constant (g mg^{-1} min^{-1}); q_t and q_e are the adsorption capacities of the ion-selective nanofibers at time t and at equilibrium respectively and t (min) is the contact time.

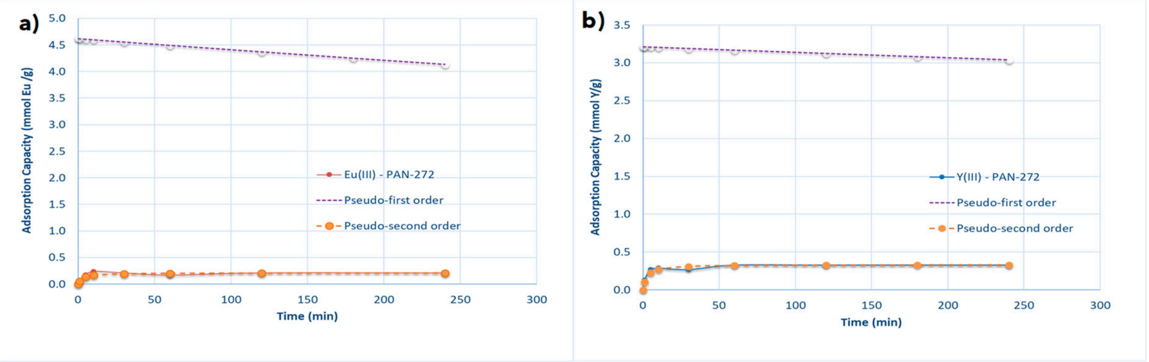

Figure S2. Fitting experimental data for first and second order kinetic models for Y(III) and Eu(III)

Langmuir and Freundlich Isotherm models

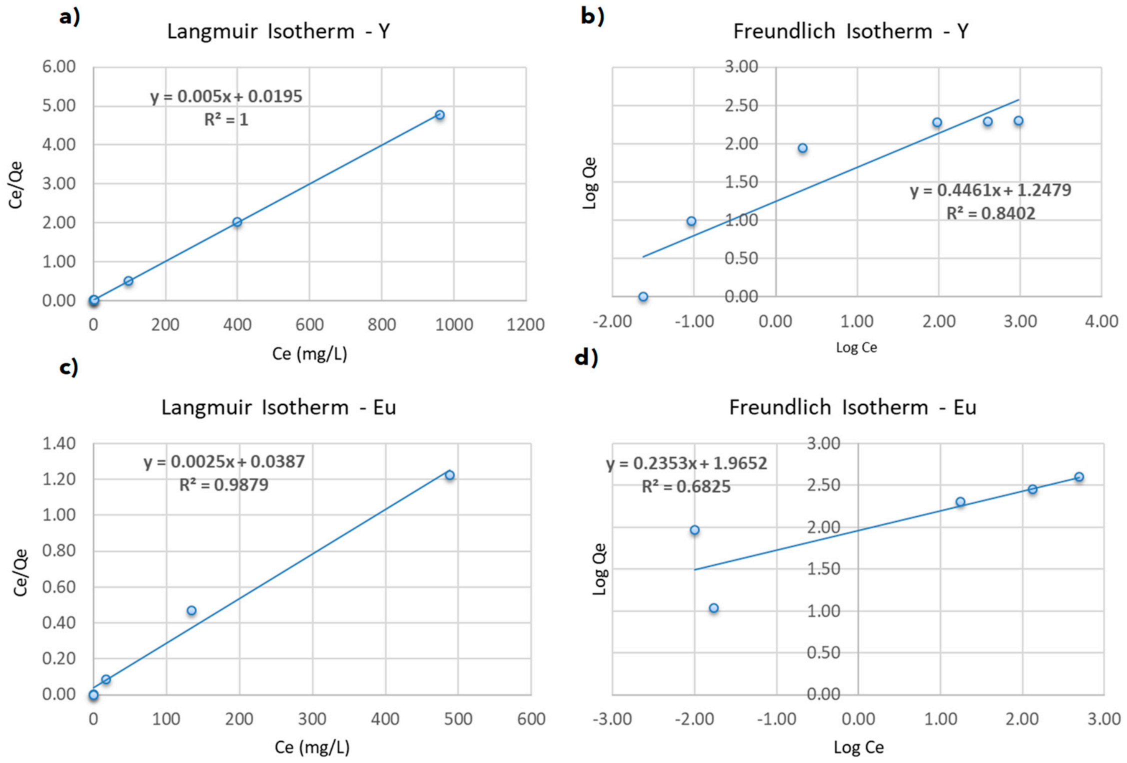

Figure S3. Fitting experimental data for Langmuir and Freundlich models for Eu(III) and Y(III)

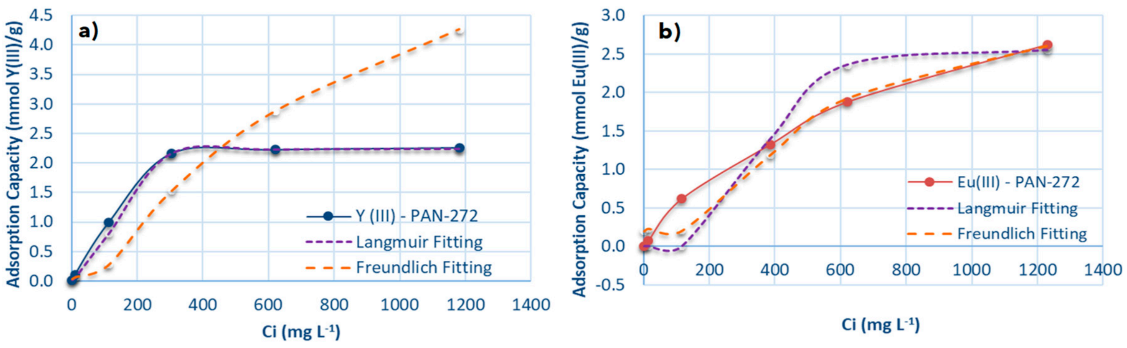

Figure S4. Langmuir and Freundlich models fitted graphs for Y(III) and Eu(III)
